# Nicotine, IFN-γ and retinoic acid mediated induction of MUC4 in pancreatic cancer requires E2F1 and STAT-1 transcription factors and utilize different signaling cascades

**DOI:** 10.1186/1476-4598-11-24

**Published:** 2012-04-26

**Authors:** Sateesh Kunigal, Moorthy P Ponnusamy, Navneet Momi, Surinder K Batra, Srikumar P Chellappan

**Affiliations:** 1 Dept. of Tumor Biology H. Lee Moffitt Cancer Center and Research Institute, 12902 Magnolia Drive, Tampa, FL 33612, USA; 2 Department of Biochemistry and Molecular Biology Eppley Cancer Institute, University of Nebraska Medical Center 985870, Nebraska Medical Center Omaha, NE 68198-5870, Nebraska, USA

**Keywords:** Mucin 4, Pancreatic cancer, Cell proliferation and invasion invasion, Src Kinase, Akt pathway.

## Abstract

**Background:**

The membrane-bound mucins are thought to play an important biological role in cell–cell and cell–matrix interactions, in cell signaling and in modulating biological properties of cancer cell. MUC4, a transmembrane mucin is overexpressed in pancreatic tumors, while remaining undetectable in the normal pancreas, thus indicating a potential role in pancreatic cancer pathogenesis. The molecular mechanisms involved in the regulation of *MUC4* gene are not yet fully understood. Smoking is strongly correlated with pancreatic cancer and in the present study; we elucidate the molecular mechanisms by which nicotine as well as agents like retinoic acid (RA) and interferon-γ (IFN-γ) induce the expression of MUC4 in pancreatic cancer cell lines CD18, CAPAN2, AsPC1 and BxPC3.

**Results:**

Chromatin immunoprecipitation assays and real-time PCR showed that transcription factors E2F1 and STAT1 can positively regulate *MUC4* expression at the transcriptional level. IFN-γ and RA could collaborate with nicotine in elevating the expression of MUC4, utilizing E2F1 and STAT1 transcription factors. Depletion of STAT1 or E2F1 abrogated the induction of MUC4; nicotine-mediated induction of MUC4 appeared to require α7-nicotinic acetylcholine receptor subunit. Further, Src and ERK family kinases also mediated the induction of MUC4, since inhibiting these signaling molecules prevented the induction of MUC4. MUC4 was also found to be necessary for the nicotine-mediated invasion of pancreatic cancer cells, suggesting that induction of MUC4 by nicotine and other agents might contribute to the genesis and progression of pancreatic cancer.

**Conclusions:**

Our studies show that agents that can promote the growth and invasion of pancreatic cancer cells induce the MUC4 gene through multiple pathways and this induction requires the transcriptional activity of E2F1 and STAT1. Further, the Src as well as ERK signaling pathways appear to be involved in the induction of this gene. It appears that targeting these signaling pathways might inhibit the expression of MUC4 and prevent the proliferation and invasion of pancreatic cancer cells.

## Background

Smoking is strongly correlated with cancers of the lung, pancreas, and prostate [1-3]. In relation to pancreatic pathology, smoking has been described as an important risk factor for chronic pancreatitis and remains the only widely acknowledged environmental risk factor for pancreatic cancer [4]. The nature of association between smoking and pancreatic cancer is, however, not yet well understood, and it remains to be elucidated whether tobacco smoke is a true etiologic factor or it helps aggravate the disease in presence of other causal risk factors [5]. Such information will provide an insight into the molecular mechanisms by which smoking accelerates the pancreatic inflammatory process and/or contributes to the pancreatic cancer development. Cigarette smoke contains a variety of chemicals, many of which are well-established carcinogens; tobacco specific nitrosamines, which are structurally related to nicotine, fall under this category [6]. Moreover, studies have shown that nicotine, the major addictive component of the tobacco smoke, induces widespread changes in the pancreatic exocrine function. Nicotine has been found to promote cell proliferation, angiogenesis as well as tumor metastasis [2,7,8], suggesting that it has the potential to act as a tumor promoter. Further, it has been reported that nicotine can prevent apoptosis induced by various chemotherapeutic agents as well as radiation, by activating various survival pathways in cancer cells [9].

MUC4, a member of the membrane-bound mucin gene family, is a high molecular weight O-glycoprotein produced by secretory epithelial cells for the lubrication and protection of ducts and lumen [10]. MUC4 is aberrantly expressed in pancreatic adenocarcinoma and tumor cell lines, while remaining undetectable in the normal pancreas or chronic pancreatitis [11]. Furthermore, a progressive increase in MUC4 expression has been observed in precancerous pancreatic intraepithelial neoplasias (PanINs) [12], indicating its role in disease development. Functional studies on MUC4 have provided substantial evidence for its role in the promotion of pancreatic cancer cell growth and metastasis [13]. Recent studies have shown that knock-down of MUC4 expression reduced pancreatic tumor cell growth and metastasis. Further the studies on Muc4 shows that it influences tumor growth via the suppression of apoptosis and potentiate metastasis via multiple mechanisms. It has been shown that overexpression of the cell-surface *Muc4*/SMC disrupts integrin-mediated cell adhesions as well as the homotypic cell-cell interactions, causing the dissociation of tumor cells in culture [14].The expression of *MUC4* can be regulated at both transcriptional and post-transcriptional levels [15,16]. There are reports showing that CDX, HNF, FOXA, GATA and HNF1α transcription factors regulate *MUC4* transcription through their binding sites present on the *MUC4* promoter [17].

Given the presence of various regulatory elements in the promoter of *MUC4*, it is not surprising that it responds to a variety of extracellular signaling molecules. Indeed, MUC4 is induced by IFN-γ as well as retinoic acid (RA) [18]. IFN-γ is a cytokine that is critical for innate and adaptive immunity against viral and intracellular bacterial infections. It is secreted by activated T lymphocytes and natural killer cells and regulates a variety of physiological responses [19] . The binding of IFN-γ to its cell surface receptor activates the receptor-associated tyrosine kinases, resulting in the activation of various STAT transcription factors and expression of their target genes [20]. Findings of Andrianifahanana et al., [21] suggest that IFN-γ can induce the expression of MUC4 through STAT1. RA is present in the plasma [22] and exerts its effects via the nuclear RA receptors and retinoic X receptors. Typically, heterodynes of RAR/RXR act as transcription factors to promote the transcription of RA-induced genes [23,24]. The multifunctional agent retinoic acid (RA) and its derivatives have been used to treat many tumor types. The antitumor effects of retinoid are in part due to their ability to inhibit proliferation of cancer cells. However, smokers receiving dietary vitamin A and beta carotene in chemoprevention studies had a higher incidence of cancer in particular pancreatic and lung cancer. These studies imply that lower doses of retinoids may have tumor-promoting activity [25]. Based on these reports we attempted to check the effect of RA on E2F1 and Stat1 transcription factor and in turn the expression of MUC4. Choudhury et al., [26] have shown that RA treatment culminated the TGF-β-2-mediated up regulation of MUC4 expression. Interestingly, IFN-γ and RA are known for their ability to evoke a synergistic effect, which leads to an enhanced induction of target gene(s) and an exacerbation of the associated biological response(s) [18]. The impact of this synergism has been observed in a wide range of malignant tumor cell types, including pancreatic tumor cells [26].

In the present study we explored the molecular mechanisms governing MUC4 expression in pancreatic cancer cell lines in response to stimulation with different agents that are known to affect the biology of pancreatic cancer. Our studies show that E2F1 and STAT1 mediate the expression of MUC4 in response to various signals and that the depletion of MUC4 prevents the proliferation and invasion of these cells in response to nicotine stimulation. These findings also reveal that different downstream signaling events mediate the induction of MUC4 in response to these agents.

## Results

### IFN-γ and RA co-operate with nicotine to induce the *MUC4* promoter

Smoking is a well-known risk factor for pancreatic cancer, while MUC4 is aberrantly over expressed in pancreatic cancer and contributes to its pathogenesis [27]. Recently, nicotine was shown to induce mucin genes in cancer [28,29] and that many endogenous molecules like Retinoic Acid (RA) [26] and IFN-γ [18] can induce expression of MUC4 in CD18/HPAF pancreatic cancer cells. Earlier studies had shown that nicotine stimulation of non-small cell lung cancer cells leads to an induction of E2F1 binding to promoters followed by their transcriptional activation [7,30]. An examination of the *MUC4* promoter showed the presence of four E2F binding sites at positions (-346 to ‐ 362, -349 to ‐ 365, -409 to ‐ 425 and -410 to - 426). Given that nicotine stimulates the binding of E2F1 to a variety of promoters, and since STAT1 is known to induce *MUC4*, we decided to examine whether these factors mediate the induction of MUC4 in pancreatic cancer cells. To examine whether E2F1 and STAT1 can bind to the *MUC4* promoter and whether such an association is induced by nicotine IFN-γ and RA, a series of chromatin immunoprecipitation experiments were carried out on four pancreatic cancer cell lines, namely CD-18/HPAF, ASPC-1, CAPAN-2 and SW1990. CD18 is a poorly differentiated cell line derived from HPAF has mutated K-Ras gene and deletions of the p53 gene; Rb-1 gene is wild type. AsPC1 is a poorly differentiated human pancreatic adenocarcinoma cell line has the mutated K-Ras, p53 and p16 genes and deletion of BRCA2 gene and wild type Rb-1. SW1990 is a well differentiated human pancreatic adenocarcinoma with K-ras mutation. CAPAN2, a moderately differentiated human pancreatic adenocarcinoma cell line has the mutated K-Ras gene and deletions of the p53 gene [31].

**Figure 1:**
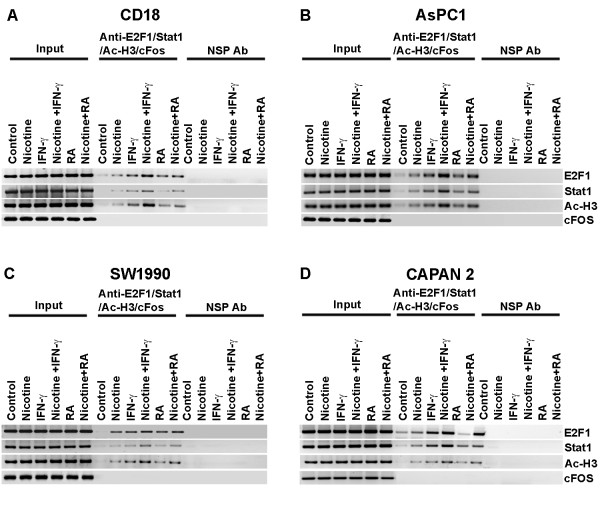
**IFN-γ and (RA) co-operate with nicotine to induce the*MUC4*promoter.** Chromatin IP assays showing the occupancy of E2F1 and STAT1 on the *MUC4* promoter in 4 different pancreatic cancer lines. CD18/HPAF-SF (**A**), ASPC-1 (**B**), SW1990 (**C**) and CAPAN2 (**D).** Cells were treated with nicotine, IFN-γ, IFN-γ in combination with nicotine, RA and RA in combination with nicotine showed increased E2F1 and STAT1 binding on the *MUC4* promoter. Sonicated genomic DNA is used for input. C-Fos was used as a negative control. Nonspecific IgG was used as a negative control in pull-down assays.

PC cells were rendered quiescent by serum starvation and stimulated with nicotine, IFN-γ alone, nicotine in combination with IFN-γ, RA alone and nicotine in combination with RA, respectively for 48 h. ChIP assay lysates were prepared using our published protocols [29,32] and immunoprecipitated with antibodies against E2F1, STAT1 as well as with an irrelevant antibody as control. It was found that there were minimal amounts of E2F1 or STAT1 associated with the *MUC4* promoter in quiescent CD18/HPAF cells. Stimulation with nicotine, IFN-γ or RA induced the binding of both E2F1 and STAT1 to the promoter (Figure 1A-D). When the cells were stimulated with a combination of nicotine with IFN-γ, there appeared to be a synergistic binding of the two factors to the promoter; in contrast, stimulation with nicotine and RA together appeared to have an added effect. There was no binding observed in lanes immunoprecipitated with the control antibody. Similar results were also obtained in other three cell lines (Figure 1A-D), but there was no noticeable co-operative effect of these agents on the association of E2F1; there appeared to be an added effect in the case of STAT1 binding in this case.

**Figure 2:**
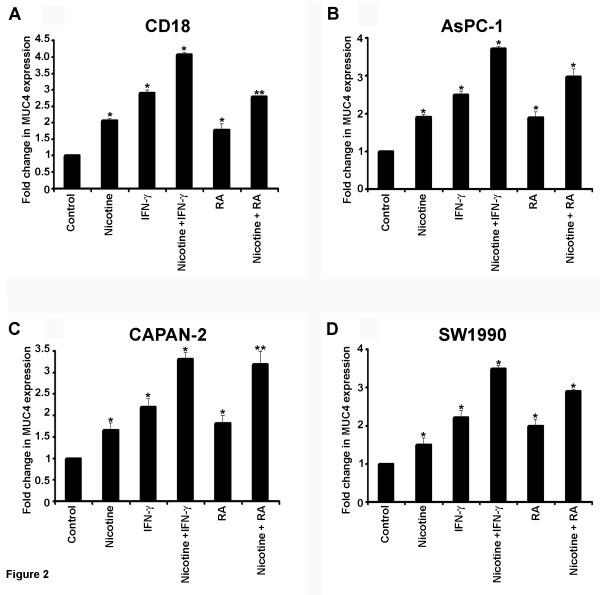
**IFN-γ and (RA) co-operate with nicotine to induce the*MUC4*promoter.** Real time-PCR showing the expression of MUC4 in CD18/HPAF (**A**), ASPC-1 (**B**), SW1990 (**C**) and CAPAN-2 (**D)** treated with nicotine, IFN-γ, IFN-γ in combination with nicotine, RA and RA in combination with nicotine. The upregulation of MUC4 upon stimulation was significant in pancreatic cancer cells treated with nicotine, IFN-γ, IFN-γ, RA or combinations (*p ≤ 0.01, **p ≤ 0.03). The results shown are the average of three separate experiments.

Transcriptional activation of genes is generally associated with acetylation of histones in their promoter region [33]. Both E2F1 and STAT1 mediated induction of transcription is known to correlate with enhanced acetylation of histones. To examine whether such an event occurs in the case of *MUC4* gene, the ChIP assay lysates were immunoprecipitated with an antibody to acetylated lysines on histone H3. As shown in Figure 1A, there was only low amount of acetylated lysines in the quiescent cells. Stimulation with nicotine, IFN-γ or RA led to a marked increase in the acetylation of lysines on the MUC4 promoter, suggesting that the promoter is transcriptionally active. Similar expression of MUC4 at protein level was confirmed by western blotting in CD18 and SW1990 cell lines (data not shown). Attempts were made to assess whether an enhanced binding of E2F1 and STAT1 correlated with elevated expression of *MUC4*. Real-time PCR assays showed that nicotine induced the expression of *MUC4* in both CD18/HPAF that produces relatively high levels of *MUC4*[26] and also in ASPC-1, CAPAN-2 and SW1990. As shown in Figure 2A-D, nicotine increased *MUC4* expression more than 2-fold in CD18/HPAF cells and nearly 2-fold in ASPC-1, CAPAN-2 and SW1990 cells compared to quiescent control cells. Further, we observed that IFN-γ and RA increased the expression of *MUC4* in CD-18/HPAF, ASPC-1, CAPAN-2 and SW1990 cells (Figure 2A). Interestingly, combination of nicotine with IFN-γ or RA led to an addictive induction of the promoter, correlating with the enhanced binding of E2F1 and STAT1 seen in ChIP assays. Taken together, these results suggest that STAT1 and E2F1 mediate the induction of *MUC4* in response to nicotine, IFN-γ and RA.

### E2F1 and STAT1 are necessary for nicotine, IFN-γ and RA-mediated MUC4 induction

**Figure 3:**
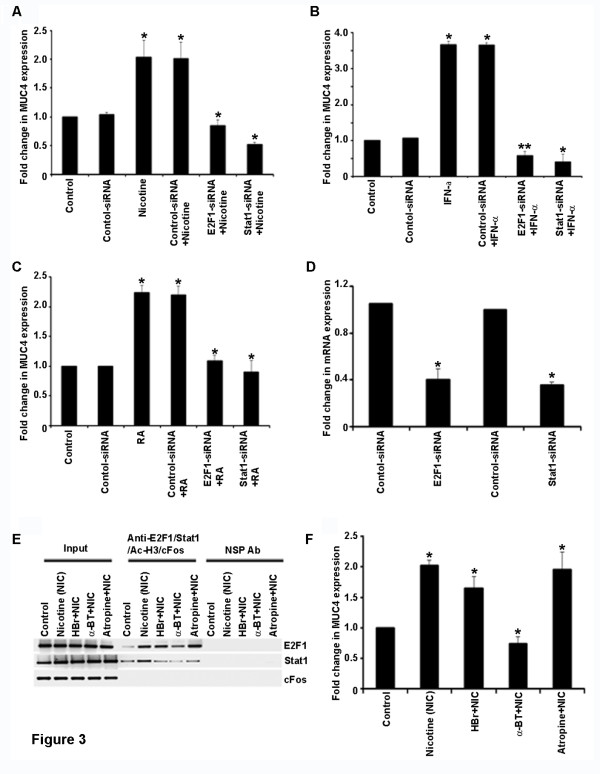
**E2F1 and STAT1 are necessary for*MUC4*induction by nicotine, IFNγ and RA.** Real time-PCR showing the expression of *MUC4* in CD18/HPAF, ASPC-1, CAPAN-2 and SW1990 pancreatic cancer cells where E2F1 and STAT1 are knocked down using respective siRNAs and subjected to nicotine stimulation (**A**). Real time-PCR showing the effect of siRNA targeting E2F1 or STAT1 on expression of Muc 4 in response to IFN-γ stimulation in CD18/HPAF pancreatic cancer cells (**B**). Real time-PCR showing the effect of siRNA targeting E2F1 or STAT1 on expression of Muc 4 in response to RA stimulation in CD18/HPAF pancreatic cancer cells (**C**). The efficiency of E2F1-siRNA and STAT1-siRNA transfection in CD18 cells is also shown by Real time-PCR (**D**). **(E)** Chip assay results suggest that α7-subunit of nAChR play an important role in mediating nicotine-induced up-regulation of *MUC4* expression in CD18**/**HPAF cells. (**F**) Real time-PCR showing the reduction in the expression of *MUC4* in CD18/HPAF cells treated with α-BT prior to nicotine stimulation. Nicotine induces MUC4 in a receptor-dependent fashion (*p ≤ 0.01, **p ≤ 0.03).

Since we found that stimulation with nicotine, IFN-γ or RA led to an increased recruitment of E2F1 and STAT1, attempts were made to see whether these transcription factors are necessary for the induction of this gene. To examine this possibility, real-time PCR experiments were conducted on cells transfected with a control siRNA or siRNA to *E2F1* or *STAT1*. Essentially, cells were transfected with the siRNAs for 24 hours and allowed to recover for 18 h. They were rendered quiescent by serum starvation and subsequently stimulated with nicotine, IFN-γ or RA for 24 h. RNA was prepared and real-time PCR was conducted using standard protocols. The efficiency of siRNA transfection was supported by real-time PCR analysis for both E2F1 and Stat1 (Figure 3D). As shown in the Figures 3A, B and C, it was found that depletion of *E2F1* or *STAT1* significantly reduced the nicotine-mediated induction of *MUC4* in CD18/HPAF cells at the transcriptional level. The results were more obvious in IFN-γ stimulation, where the induction was completely inhibited when these factors were depleted (Figure 3B). Similarly, RA stimulation required both these factors in CD18/HPAF cells (Figure 3C). Given that *E2F1* siRNA and *STAT1* siRNA reduces the expression of these transcription factors as expected (Figure 3D), these results in combination with the ChIP assay results, strongly suggest that E2F1 and STAT1 play a major role in mediating the induction of the *MUC4* gene in pancreatic cancer cells in response to various upstream signals.

### Nicotine induces MUC4 in a receptor-dependent fashion

Nicotine exerts its biological effects through nicotinic acetylcholine receptors (nAChRs) that are widely expressed in neurons and at neuromuscular junctions; they are present on a wide array of non-neuronal cells as well. We next examined whether nicotine-mediated recruitment of E2F1 and STAT1 on the *MUC4* promoter required nAChR function. Towards this purpose, quiescent CD18/HPAF cells were stimulated with nicotine in the presence of hexamethonium bromide or α-bungaratoxin, which are nAChR antagonists; atropine, which is an antagonist of muscarinic acetylcholine receptors, was used as a control. ChIP assay results suggests that α-bungarotoxin sensitive α7 nAChR subunit plays an important role in mediating nicotine-induced recruitment of E2F1 and STAT1 to the *MUC4* promoter, since cells treated with this agent showed lower amounts of E2F1 and STAT1 on the *MUC4* promoter (Figure 3E). On the other hand, cells treated with atropine showed no reduction in the recruitment of these factors, suggesting that muscarinic type acetylcholine receptors play no role in the recruitment of these regulatory factors.

Experiments were conducted to assess whether the transcriptional induction of *MUC4* correlated with the enhanced binding of these factors and whether nAChR antagonists had a similar effect. Real-time PCR experiments were conducted on CD18/HPAF cells treated with hexamethonium bromide, α-BT or atropine and stimulated with nicotine. The induction of *MUC4* was assessed by real-time PCR. As shown in Figure 3F, stimulation with nicotine induced *MUC4* promoter in CD18 cells; the stimulation was abrogated in the presence of hexamethonium bromide and α-BT, but not atropine. These results suggest that nAChRs, especially the α7 subunit, plays a major role in nicotine-mediated stimulation of the *MUC4* gene.

### Differential contribution of Akt, Src and ERK signaling in regulating MUC4 expression

**Figure 4:**
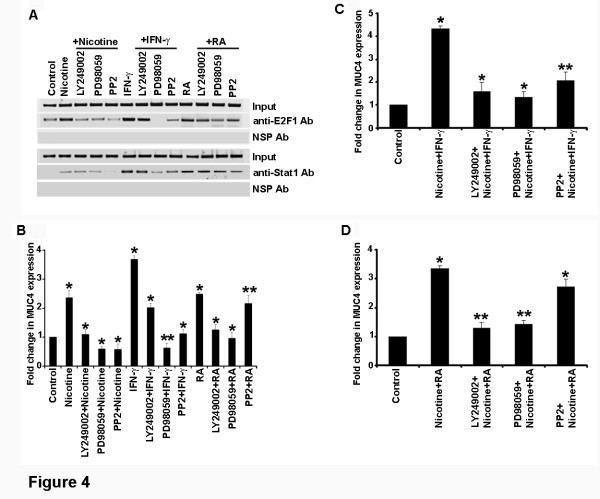
**Differential contributions of Akt, Src and ERK signaling in regulating*MUC4*expression.** (**A**) ChIP assay conducted on CD18 cells stimulated with nicotine, IFN-γ or RA in the presence of chemical inhibitors of LY294002, PD98059 and PP2 showed ERK and Src-family kinases may be involved in the upregulation of *MUC4* upon nicotine stimulation. At the same time, in the case of IFN-γ LY294002, PD98059 and PP2 showed significant decreased expression of MUC4, whereas with RA stimulation LY294002 and PD98059 showed decreased expression of MUC4 but PP2 did not show significant inhibition in the expression of MUC4. (**B**) Real time-PCR supported the ChIP assay results where ERK and Src-family kinases were involved in the upregulation of *MUC4* upon nicotine stimulation. In the case of IFN-γ, PI3K, MEK and Src family kinases are involved in the expression of MUC4, whereas with RA stimulation PI3K and MEK kinases are involved in the expression but Src-family kinases had a lesser role. (**C**) Real time-PCR showing combination of nicotine and IFN-γ involves ERK as well as Src in the induction of the *MUC4* promoter whereas (**D**) Src seemed to have only a minimal effect when RA was combined with nicotine (*p ≤ 0.01, **p ≤ 0.03).

Experiments were conducted to understand the downstream signaling events that mediate the induction of MUC4 in response to nicotine, IFN-γ and RA stimulation. We focused on Akt, Src and Erk pathways, since they are known to mediate the effects of nicotine in different systems. In this initial set of experiments, ChIP assays were conducted on quiescent CD18 cells or those stimulated with nicotine, IFN-γ or RA alone, or in the presence of LY249002, a PI3 kinase inhibitor, or PD98059, a MEK inhibitor or PP2, a Src kinase inhibitor. It was found that nicotine-mediated recruitment of E2F1 and STAT1 required signaling through all the three pathways tested (Figure 4A); Src seemed especially vital for the enhanced association of STAT1 with the promoter. In contrast, IFN-γ stimulation did not require PI3 kinase/Akt pathway to recruit E2F1 or STAT1, but ERK and Src seemed to contribute. In the case of RA stimulation, the contribution of Src seemed minimal, while Akt and ERK pathways appeared to be important. The signaling requirements were similar in both the cell lines tested.

Real-time PCR assays were conducted to assess whether the requirement of E2F1 and STAT1 observed with the inhibitors correlated with the expression of the *MUC4* gene as well. As shown in Figure 4B, it was found that the expression pattern paralleled the binding of E2F1 and STAT1; thus, nicotine stimulation required mainly ERK and Src pathways, while IFN-γ required the contribution of all the three pathways to a certain extent. One point of variation was the contribution of the PI3K/Akt pathway, which had minimal impact on the recruitment of E2F1 and STAT1, but had significant impact on gene expression. In the case of RA stimulation, the main contributors were PI3 Kinase/Akt pathway as well as ERK pathway, with Src playing a minimal role. These studies show that *MUC4* gene can respond to various signaling pathways induced by different upstream molecules.

Real-time PCR experiments were also conducted to assess whether the same pathways are operational when two of the stimulatory agents are used in combination. As shown in Figure 4C-D the PI3/Akt, ERK as well as Src seemed to be involved in the induction of the *MUC4* promoter when nicotine and IFN-γ was used in combination. Similarly, Src seemed to have only a minimal effect when RA was combined with nicotine. These results show that the major mediators of MUC4 induction are PI3K/Akt, ERK and Src kinases, depending on the upstream activation agents.

### Involvement of JAK-STAT signaling in upregulation of MUC4

**Figure 5:**
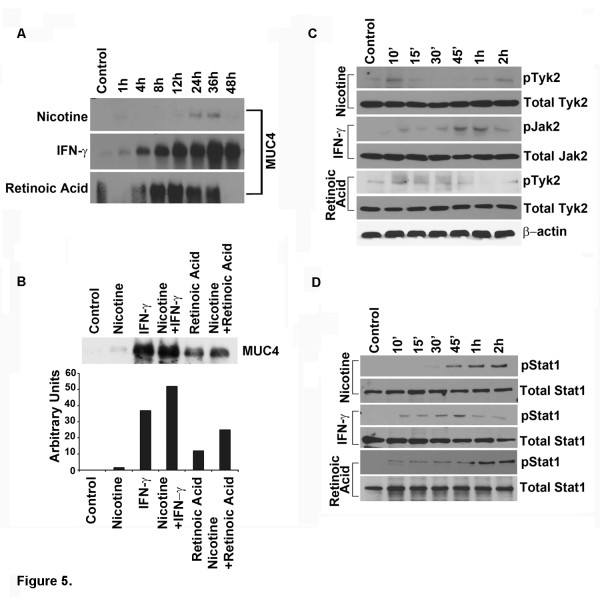
**Involvement of Jak-Stat signaling in upregulation of MUC4.** (**A**) The expression of MUC4 in CD18 cells upon treatment with Nicotine, IFN-γ and RA were analyzed by agarose gel electrophoresis. Serum-starved CD18 cells were treated with 1 μM nicotine, IFN-γ and RA for the given time points. (**B**) MUC4 expression at protein level was analyzed in nicotine in combination of IFN-γ and also nicotine in combination with retinoic acid by western blot analysis and the quantification of the bands is shown below. (**C**) Kinetics (starting at 10 min - 2 h) of Phosphorylation status of Jak kinases at the protein level was analyzed by immunoblotting. (**D**) Kinetics (starting at 10 min - 2 h) of Phosphorylation status of Stat1 at the protein level was analyzed by immunoblotting. In addition, the levels of total Tyk2, Jak2 and Stat1 were also assessed by immunoblotting. β-actin was used as a loading control. All immunoblotting results are representative of two independent experiments.

Expression of MUC4 at protein level increased at 24 h in Nicotine and after 4 h in IFN-γ and RA treatment as shown by SDS-Agarose gel electrophoresis (Figure 5A). Further, we found that the expression of MUC4 was more than 8 fold in IFN-γ treated cells compared to the control cells and more than 3 fold in RA treated cells. Furthermore the expression of MUC4 in nicotine and IFN-γ treated cells was nearly one and half fold more than IFN-γ alone and nearly 0.5 fold more in nicotine and retinoic acid than retinoic acid alone treated CD18 cells (Figure 5B). A time dependent treatment with nicotine, IFN-γ and Retinoic acid showed a gradual increase in the phosphorylation of Tyk2 and Stat1 in the HPAF/CD18-SF cells (Figure 5C). 1 μM nicotine showed a slight increase in the Tyk2 and Stat1 phosphorylation in CD18 cells at 10-15 min and 30-45 minutes respectively (Figure 5D), whereas, no change was observed in the total Tyk2 and Stat1 expression. We also checked for the different Jak kinase family members but we did not see any change in the phosphorylation status of other family members (data not shown). These results suggest that Tyk2 and STAT1 contribute to the induction of MUC4 in response to various signals.

### MUC4 is necessary for nicotine-induced proliferation and invasion of pancreatic cancer cells

**Figure 6:**
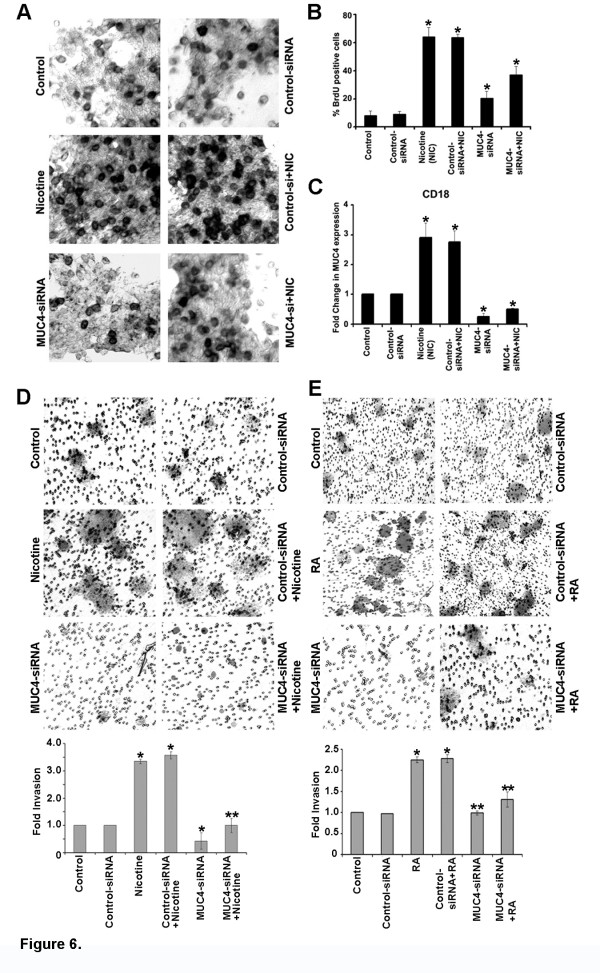
**Nicotine induces proliferation and invasion of pancreatic cancer cells.** (**A**) Quiescent CD18 cells were stimulated with 1 μM nicotine for 18 h and S-phase entry was measured by BrdU assays. The proliferative effects of nicotine in pancreatic cancer cells were abrogated in the MUC4 silenced cells, indicating that MUC4 function is required for the proliferative effects of nicotine. (**B**) Shows the efficiency of *MUC4*-siRNA transfection in CD18 cells. (**C**) Quantification of proliferation assay. (**D**) Nicotine was able to potently promote invasion of CD18 cells at a concentration of 1 μM as seen in a Boyden-chamber assay. The pro-invasive activity of nicotine was abrogated by *MUC4*-siRNA demonstrating a requirement for MUC4 role in invasion. Graphical representation of the results from Boyden-chamber assay shows the results are significant (*p ≤ 0.01, **p ≤ 0.04). (**E***)* RA was able to potently promote invasion of CD18 cells at a concentration of 10 nM as seen in a Boyden-chamber assay. The pro-invasive activity of RA was abrogated by *MUC4*-siRNA significantly demonstrating a requirement for MUC4 role in invasion. Graphical representation of the results from Boyden-chamber assay (*p ≤ 0.01, **p ≤ 0.03).

Fauquette et al. [34-36] has reported that MUC4 plays a pivotal role in the proliferation and invasion of pancreatic cancer cell lines. Our earlier experiments had shown that nicotine promotes the proliferation as well as invasion of a variety of lung cancer cell lines and that nicotine enhances metastasis in mouse models of lung cancer [2]. Given this background, experiments were conducted to assess whether MUC4 plays a role in mediating the proliferation as well as invasion of pancreatic cancer cells. In the first set of experiments, CD18/HPAF cells were transfected with a control siRNA or siRNA to *MUC4*; cells were rendered quiescent by serum starvation for 18 h and stimulated with nicotine for 24 h. Cell proliferation was assessed by measuring BrdU incorporation, using the kit according to the manufacturer’s protocol. It was found that depletion of MUC4 greatly reduced the proliferation of both CD18 cells when stimulated with nicotine (Figure 6A-C). Similar results were obtained when a different siRNA to MUC4 was used (data not shown). This result clearly shows that MUC4 is a major mediator of the proliferative effects of nicotine. IFN-γ and RA did not have a significant proliferative effect on the cells and were not studied further.

Boyden chamber assays were carried out to assess whether MUC4 play a role in nicotine-mediated invasion of pancreatic cancer cells. As in the previous experiments, CD18 cells were transfected with a control siRNA or siRNA to *MUC4* and serum starved for 18 h. Cells were stimulated with nicotine and plated on Boyden chambers. Invading cells could be visualized using crystal violet staining of the membranes (Figure 6D). It was found that depletion of *MUC4* greatly inhibited the invasive properties of both the cell lines. The results are depicted graphically in Figure 6B; these results were confirmed by using a different siRNA to MUC4 and similar results were obtained (data not shown). These studies show that MUC4 is a major mediator of nicotine functions and is involved in promoting proliferation as well as invasion of pancreatic cancer cells. Figure 6E, shows that RA stimulated cells have invasive properties similar to nicotine stimulated cells but this is significantly inhibited by the depletion of MUC4 in CD18 cells. But IFN-γ did not have any significant effect on the invasive behavior of CD18 cells.

## Discussion

Understanding of molecular mechanisms that govern tissue-specific gene expression often lead to the identification of transcription factors responsible for overexpression of certain genes leading to tissue specialization and maturation. In this report, we show that E2F1 and STAT1 are activators of MUC4 mucin tumor marker. We find a positive correlation between the binding of E2F1 and STAT1 with *MUC4* promoter and its expression in pancreatic cancer cell lines. As reported in other studies, MUC4 is expressed in 83 % of pancreatic ductal adenocarcinoma samples, both poorly differentiated as well as well-differentiated types [34]. No expression was found in normal pancreas or chronic pancreatitis [37]. The significant overexpression of MUC4 points to an important role for MUC4 in tumor progression, especially in pancreatic cancer. However, the molecular mechanisms underlying the dysregulation of MUC4 observed in pancreatic cancer are still poorly understood. In this paper, we investigated the role of E2F1 and STAT1 transcription factors on MUC4 regulation in pancreatic cancer cells and found that both the transcription factors can positively regulate *MUC4* transcription. The results obtained at the promoter level correlate well with those obtained at the mRNA level, in response to three different extracellular signals.

The biological effects of nicotine are mediated by nAChRs, which are widely expressed in neurons and neuromuscular junctions; certain subtypes of the receptor are expressed on a variety of non-neuronal cells as well. Recent reports show that cigarette smoke ingredients can modulate the α7 and α4β2 nAChRs and has shown the presence of these receptors on lung and pancreatic cancer cells [2,38]. Attempts made to elucidate the increased recruitment of E2F1 and STAT1 in response to nicotine stimulation showed a requirement of the α7 subunit. This was determined using specific antagonists of the α7-subunit (α-bungarotoxin), which blocked nicotine-mediated recruitment of the transcription factor on to the *MUC4* promoter. Apart from this, the Real-time PCR results showed that the expression of *MUC4* upon nicotine stimulation was significantly suppressed by α-bungarotoxin. These results suggest that the increased expression of MUC4 by nicotine is mediated through α7-subunits nAChRs on pancreatic cancer cells. Earlier studies had shown that different subunits mediate the proliferative and survival functions of nicotine in lung cancer cells [2,7,9,30]; it appears that α7, which is more relevant to cell proliferation, mediates the induction of MUC4 in these experiments.

**Figure 7:**
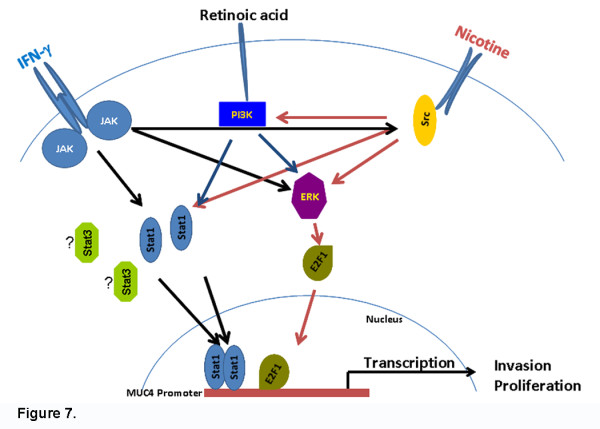
**Schematic representation of signaling involved in MUC4 expression upon Nicotine, IFN-γ and Retinoic acid stimulation.** Nicotine-mediated recruitment of E2F1 and STAT1 requires signaling through all the three pathways tested. In contrast, IFN-γ stimulation did not require PI3 kinase/Akt pathway to recruit E2F1 or STAT1, but ERK and Src seemed to contribute. In the case of RA stimulation, Akt and ERK pathways appeared to be important in upregulation of MUC4 expression.

The proto-oncogene c-Src is a non-receptor tyrosine kinase whose expression is correlated with cancer progression and poor prognosis in pancreatic cancer. Src family kinases are involved in regulating signaling of receptor tyrosine kinases, G-protein-coupled receptors and FAK influencing wide array of functionalities of tumor cell behavior like proliferation, survival, angiogenesis, adhesion, invasion, and metastasis [39,40]. Src integrates divergent signals, facilitating the action of other signaling proteins; it is able to channel phosphorylation signals through Ras/Raf/ERK1/2 and also PI3-K/AKT pathways [41,42]. Attempts were made to understand the molecular mechanisms underlying the overexpression of MUC4 by nicotine, IFN-γ and RA. It is well documented that nicotine stimulates phosphorylation and activation of ERK1/2 [43]; the Akt pathway has been implicated in nicotine function for cell survival [9] and our lab reported that nicotine activates Src kinase [7]. ChIP assays as well as the real-time PCR results showed that the ERK and Src-family kinases are involved in the upregulation of MUC4 upon nicotine stimulation. At the same time in the case of IFN-γ stimulation, all the three inhibitors (LY294002, PD98059 and PP2) showed a decreased expression of MUC4 whereas with RA stimulation, PP2 did not show a significant inhibition in the expression of MUC4. This suggests that the PI3 kinase pathway plays a role in IFN-γ and RA-mediated induction of MUC4, but not a major role in nicotine-mediated stimulation of this promoter. It thus appears that different signaling components mediate the induction of MUC4 in pancreatic cancer cells depending upon the stimulant. While these signaling molecules facilitate nicotine stimulated induction of MUC4, it is likely that other kinases like the JAK family proteins might also contribute to the induction. These JAK kinases are known to modulate multiple STAT family members, including STAT1 and STAT3.These members of the signal transducer and activator of transcription (STAT) family of transcription factors have been implicated in transformation, tumor cell survival, invasion, and metastasis. Hence role of additional STAT family members cannot be ruled out. A schematic of the signaling pathways involved in the induction of MUC4 is shown in Figure 7.

The E2F transcription factors play a role in diverse biological functions such as cell proliferation, differentiation and apoptosis. Studies presented here show that it may also regulate the expression of genes like *MUC4*, which contribute to oncogenesis and tumor progression. Interestingly, E2F1 and STAT proteins appear to contribute to the induction of MUC4 in response to multiple signals, including the major addictive component of cigarette smoke. Our results show that nicotine-induced MUC4 can promote the proliferation and invasion of pancreatic cancer cells, whereas, RA-induced MUC4 can promote invasion but not proliferation.

## Conclusions

These studies demonstrate that E2F1 and STAT1 transcription factors play an important role in the regulation of *MUC4* gene transcription in pancreatic cancer cells. Our findings will lead to a better understanding of the mechanisms leading to the aberrant expression of MUC4 in pancreatic cancer cell lines. Additionally, this study reveals the complexity involved in the regulation of *MUC4* promoter and shows that this process may involve many signaling pathways and transcription factors that might mediate the over expression of MUC4 in pancreatic cancer.

## Methods

### Cell culture

CD18, CAPAN-2 and SW1990 pancreatic cancer cell lines were cultured in DMEM (Mediatech Cellgro, Manassas, VA) containing 10 % FBS (HyClone, Logan, UT) and ASPC-1 was cultured in RPMI1640 containing 10 % FBS. All reagents for cell culture were purchased from Invitrogen (Carlsbad, CA, USA). IFN- γ (50 ng) was obtained from Peprotech (Rocky Hill, NJ, USA). RA (10 nM) was obtained from (Sigma Chemical Company, St. Louis, MO). The studies involving signal transduction inhibitors were done on cells that were rendered quiescent by serum starvation for 24 h, following which cells were treated with indicated concentrations of the inhibitors for 30 min. Thereafter, cells were stimulated with 1 μM nicotine (Sigma Chemical Company, St. Louis, MO) in the presence or absence of the inhibitors for 48 h. The concentrations of inhibitors used for the various experiments were 1 μM PP2, 1 M atropine, 1 μM DhβE, 1 mM α-bungarotoxin and 20 μM hexamethonium bromide.

### Western Blot analysis

Cell lysates were prepared as described previously [13]. Protein concentrations were determined using a BIO-RADD/C protein estimation kit. For MUC4, the proteins (30 μg) were resolved by electrophoresis on a 2 % SDS-agarose gel under reducing conditions. Resolved proteins were transferred onto the nitrocellulose membrane and blocked in 5 % non-fat milk in phosphate buffered saline (PBS) for 1 h and subjected to the standard immunodetection procedure using specific antibodies. MUC4 immunodetection, anti-MUC4 mouse monoclonal antibody (8 G7, generated in our laboratory) in dilution of 1:1000 was used. Further, the membranes were incubated in Horseradish peroxidase-conjugated secondary antibodies (Thermoscientific, Rockford, IL) (diluted at 1:2000 in PBST) for 1 h at room temperature, followed by three washes in PBST. The blots were processed with ECL Chemiluminescence kit (GE Healthcare) and the signal was detected by exposing the processed blots to X-ray films (Biomax Films, Kodak, NY). Lysates from CD18 cells stimulated with nicotine, IFN-g and retinoic acid for different time points were prepared by Nonidet P-40 lysis as described in [44] 60 μg of total Lysates were run on 8 % SDS-polyacrylamide gel and transferred on nitrocellulose membrane by semidry method to assess the levels of Stat1 and Jak kinases by Western blotting. Actin (Sigma) was used as loading control for total lysates.

### Chromatin Immunoprecipitation (ChIP) analysis

Quiescent pancreatic cancer cell lines were stimulated with 1 μM nicotine for 24 h. A total of 2.5 × 10^7^ cells were used per immunoprecipitation (IP) reaction. Cells were crosslinked with 1 % formaldehyde for 20 min at room temperature. The crosslinking was terminated by addition of 0.125 μM glycine. Subsequently, cells were harvested and lysates were prepared [44,45]. The lysates were immunoprecipitated with polyclonal E2F1 and polyclonal STAT1 antibodies (Santa Cruz Biotechnology, Inc.). The differential binding of E2F1 and STAT1 to the region −131 to +46 (containing putative E2F1 and STAT1 binding sites) of the *MUC4* promoter was analyzed by PCR. The sequences of the PCR primers used are as follows: E2F1 (region −131 to +46) forward primer, 5′-CGCCTCTACTCCCAGAAG-3′; E2F1 (region −131 to +46) reverse primer, 5′ -TGTAGAGATGCGGTGGTC-3′; STAT1 (region −920 to −773) forward primer, 5′-CCAAAGCAGAGGACACAC-3′.

### Real-time PCR analysis

Real-time PCR was performed in a total volume of 25 μl using qPCR-Master-Mix-plus-dNTP kit (BioRad, USA) and analyzed on a BioRad Real-Time PCR system (BioRad, USA). A 1 μl of cDNA per sample was used as template. All amplifications were performed in triplicates. The thermal cycling conditions included 50°C for 2 min and 95°C for 10 min, followed by 40 cycles of 95°C for 15 s and 60°C for 1 min.

### Primers and probes

Primers and probe sets for *MUC4* were sourced from published reports [46] and synthesized by IDT DNA Technologies. A short 82 bp fragment of *MUC4* at its 3’ end was amplified using a forward primer (5’-TGGACATGCGGGCCTTT-3’) binding in exon 22 and a reverse primer (5’-GGCGGTGCTGCAGAA-3’) binding in exon 23 of full-length *MUC4*. The endogenous human glyceraldehyde-3-phosphate dehydrogenase (*GAPDH*) was used as control.

### Matrigel invasion assay

The invasive ability of CD18 cells was assayed according to the method reported before [2] . Briefly, the upper surface of the filters was precoated with collagen (100 μg / filter). Matrigel was applied to the upper surface of the filters (50 μg/ filter) and dried in a hood. These filters were placed in Boyden chambers. Cells were grown to 70 % confluency in respective media and were rendered quiescent by serum starvation, then treated with 1 μM nicotine in the presence or absence of indicated inhibitors for 18 h. Following treatment, cells were trypsinized and 10,000 cells were plated in the upper chamber of the filter in media containing 0.1 % bovine serum albumin (Sigma Chemical Company, St. Louis, MO), inhibitors and nicotine. Media containing 20 % fetal bovine serum was placed in the lower well as a chemo-attractant, and the chambers were incubated at 37°C. After 36-48 h, nonmigrating cells on the upper surface of the filters were removed by wiping with cotton swabs. The filters were processed first by fixing in methanol followed by staining with crystal violet. The cells migrating on the other side of the filters were quantitated by counting 3 different fields under 40X magnification. Data presented is a mean of 3 independent experiments.

### Proliferation assays

Bromodeoxyuridine (BrdU) labeling kits were obtained from Roche Biochemicals, Indianapolis, IN and proliferation assay was performed as described earlier [47]. Briefly, cells were plated in poly-D-lysine coated chamber slides at a density of 10,000 cells per well and rendered quiescent by serum starvation for 24 h. Cells were then stimulated with 1 μ M nicotine, IFN-γ or RA for 18 h. S-phase cells were visualized by microscopy and quantitated by counting 3 fields of 100 cells in quadruplicate. Data is presented as the percentage of BrdU positive cells out of the 100 cells counted.

### Statistical analysis

Statistical analysis was conducted using Student t test. Values were considered significant when *p* was less than 0.05

## Abbreviations

IFN-γ = Interferon-γ; RA = Retinoic acid; RAR = Retinoic acid receptor; STAT1 = Signal transducer and activator of transcription 1.

## Competing interests

The authors have no competing interests to declare.

## Authors’ contributions

SK conducted the experiments and drafted the initial manuscript; MPP and NM contributed to the experiments; SKB was involved in designing the experiments and editing the manuscript; SPC directed the study and was involved in writing the manuscript. All authors read and approved the final manuscript.
